# Supplementary material for: Enhancement of the performance of wireless sensor networks using the multihop multiantenna power beacon path selection method in intelligent structures
Source: PLoS One. 2022 Nov 7;17(11):e0276940. doi: 10.1371/journal.pone.0276940 (PMC9639829; doi:10.1371/journal.pone.0276940)

The points extracted from images for analysis.

- Fig 3, OP as a function of transmitting power P in (db)


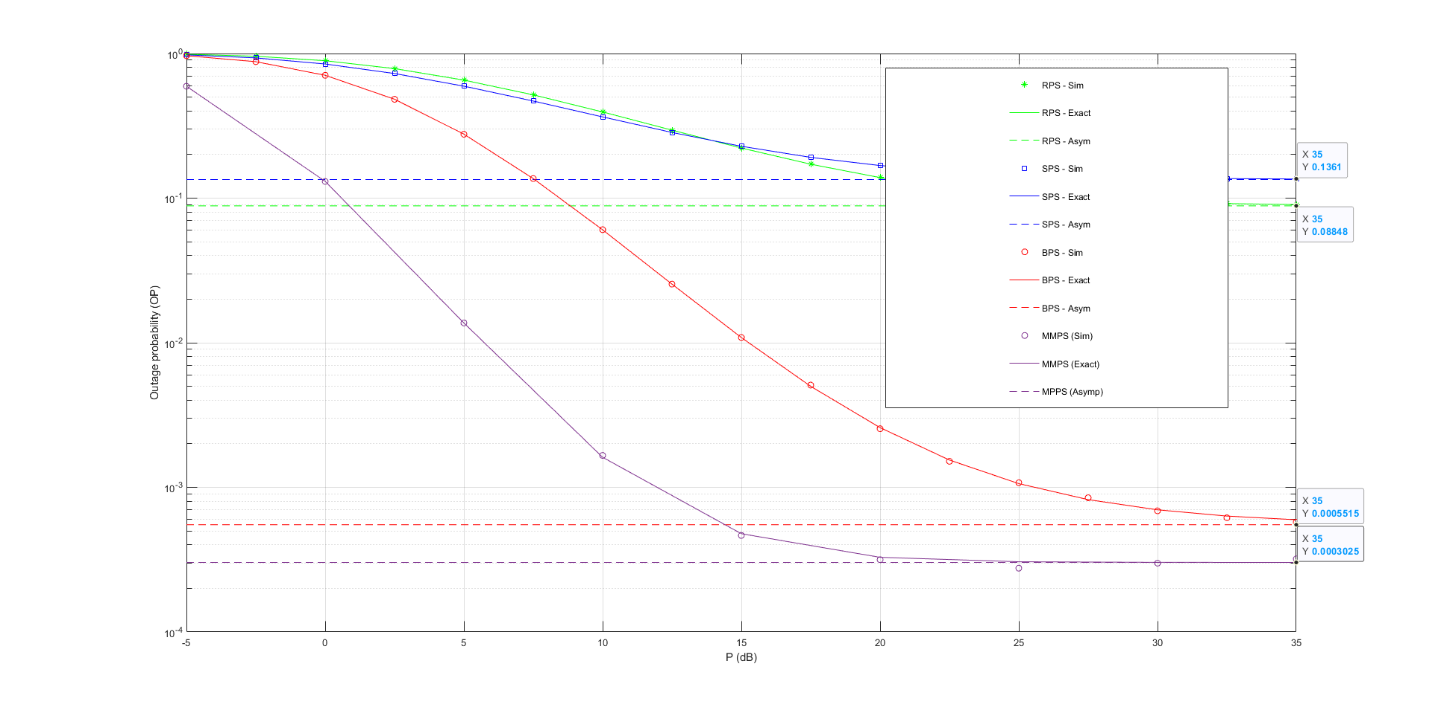


- Fig 4, OP as a function of level of impairments


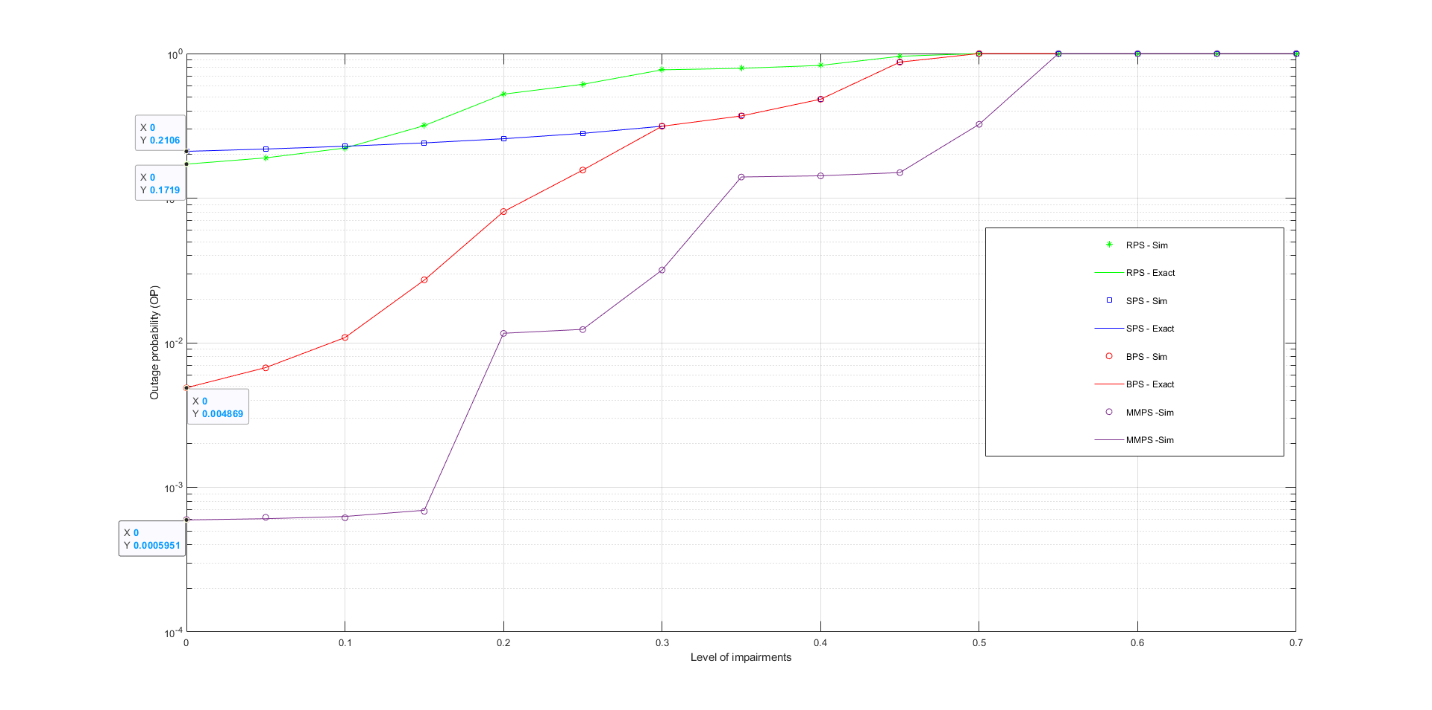

Supplement: S1 File — (DOCX) [file pone.0276940.s001.docx]
