# Supplementary material for: Enhancement of the performance of wireless sensor networks using the multihop multiantenna power beacon path selection method in intelligent structures
Source: PLoS One. 2022 Nov 7;17(11):e0276940. doi: 10.1371/journal.pone.0276940 (PMC9639829; doi:10.1371/journal.pone.0276940)
Supplement: Attachment — Submitted filename: Response to Reviewers.docx [file pone.0276940.s002.docx]

Date: October 5, 2022

**Response letter for the manuscript editor**

**Dear professor** Dr Solna Carreon Santos

Editorial Department

Journal of PLOS ONE

**Subject**: **Submission of revised paper manuscript**

We authors would like to acknowledgement the reviewers for their critical looking at the article and constructive comments for possible publication. We also appreciate the editor for your great contribution and considered our manuscript entitled “**Enhancement of the Performance of Wireless Sensor Networks Using the Multihop Multiantenna Power Beacon Path Selection Method in Intelligent Structures”** (PONE-D-22-22300R1) for possible publication if the comments are well addressed and satisfied the editors and reviewers. We have carefully reviewed the comments and have revised the manuscript and abstract accordingly. Our responses are given in a point-by-point as comment and its response. We tried to address all the specific comments from the editor and reviewers. We hope the revised version is now suitable for publication and look forward to hearing from you in due course. If you need further information about it, we are waiting for you.

Sincerely

Ahmed Hammad

Department of Electronics and Communications Engineering, Mansoura University, Mansoura, Egypt P.O. Box 35516, email, ahmed_khairt@std.mans.edu.eg

On the behalf of co-authors

**Comments from Editor**

We confirm that the submission contains "minimal data set”, which PLOS defines as consisting of the data set used to reach the conclusions drawn in the manuscript with related metadata and methods, and any additional data required to replicate the reported study findings in their entirety. This includes:

1) The values behind the means, standard deviations and other measures reported. These values are shown in detail in the manuscript.

2) The values used to build graphs. These values found in Table 1 and Table 2.

3) The points extracted from images for analysis.

- Fig 3, OP as a function of transmitting power P in (db)

- Fig 4, OP as a function of level of impairments

**Comments from Reviewer 1**

**Comment:** The abstract should be rewritten. The results should be briefly stated in the abstract.

**Response:** Thank you for pointing this out. We agree with this comment. Therefore, we have rewritten the abstract.

**Comment:** The keyword should be written.

**Response:** Agree. We have written the keyword to emphasize this point.

**Comment:** The motive of the proposed method is not clear. The motivation of the proposed method should be stated in the introduction.

**Response:** We agree with this and have incorporated your suggestion throughout the manuscript in section II named by Motivations and related work.

**Comment:** Related work has not been written. In the RELATED WORK section should focus more on differences between this paper and other works to highlight the novelty of this paper. Also, the disadvantages and shortcomings of the previous methods that are addressed in the proposed method must be stated.

**Response:** We agree with this and have incorporated your suggestion throughout the manuscript in section II named by Motivations and related work.

**Comment:** More achievements on this topic should be added for the Section "introductions" and "Related work".

**Response:** We agree with this and have incorporated your suggestion throughout the manuscript.

**Comment:** Some references are missed. At the same time, many important recent references are missing, which can support the idea of this paper, the following references should be added in the Section "References"

**Response:** We agree with this and have incorporated your suggestion throughout the manuscript.

**Comment:** In the Performance Evaluation section, specify what methods are compared with the proposed method.

**Response:** Agree. We have done to emphasize this point.

**Comment:** Mathematics modelling to analyze the method is not enough. The relative key equations should be embedded into the algorithms.

**Response:** You have raised an important point here. However, we believe that most of relative key equations are embedded into the algorithm, because the references added for the compared algorithms have all the required equations.

**Comment:** The parameter of each equation must be described after using it. The parameters of some equations are not described.

**Response:** Agree. We have done to emphasize this point.

**Comments from Reviewer 2**

**Comment:** 1. Page 1- Abstract. What is equipment malfunction? Please elaborate.

**Response:** Most of published publications on performance evaluation make the transceiver hardware in wireless terminals perfect assumptions. However, in reality, it is suffered from phase noises, amplifier-amplitude non-linearity, and in phase and quadrature imbalance (IQI), all of which considerably worsen the performance of wireless communication systems.

**Comment:** 2. Page 3- Significant Contributions. Authors are required to provide a brief summary of the stated contribution: “Using not cooperate eavesdropping scenario”.

**Response:** Agree. We have done to emphasize this point.

**Comment:** 3. Page 1- Abstract. The proposed protocol improves the protection for multi-hop uncooperative wireless sensor networks. Authors need to provide an explanation for the “uncooperative wireless sensor networks”. What is the significance of multi-hop uncooperative wireless sensor networks in context of proposed research?

**Response:** We use a non-cooperative eavesdropping scenario, where eavesdroppers don’t work as a team, but they work individually.

**Comment:** 4. Page 8-Performance Evaluation. Authors have used Monte-Carlo simulation to evaluate the performance of proposed protocol. Authors include a few references or citations regarding the Monte Carlo simulation.

**Response:** Agree. We have done to emphasize this point.

**Comment:** 5. Authors can add a separate section of the “Related Works” to investigate the recent published work for proposing the approach.

**Response:** Agree. We have done to emphasize this point.

**Comment:** 6. How does the multi-hop, multi-path wireless sensor network protect against eavesdropping? Show with the help of security analysis.

**Response:** Agree. We have done to emphasize this poin
